# Thermostable DNA ligases from hyperthermophiles in biotechnology

**DOI:** 10.3389/fmicb.2023.1198784

**Published:** 2023-05-24

**Authors:** Jingru Shi, Philippe M. Oger, Peng Cao, Likui Zhang

**Affiliations:** ^1^College of Environmental Science and Engineering, Yangzhou University, Yangzhou, China; ^2^University of Lyon, INSA de Lyon, CNRS UMR, Villeurbanne, France; ^3^Faculty of Environment and Life, Beijing University of Technology, Beijing, China

**Keywords:** DNA ligase, ligase cycling reaction, DNA manipulation, thermostability, hyperthermophiles

## Abstract

DNA ligase is an important enzyme ubiquitous in all three kingdoms of life that can ligate DNA strands, thus playing essential roles in DNA replication, repair and recombination *in vivo*. *In vitro*, DNA ligase is also used in biotechnological applications requiring in DNA manipulation, including molecular cloning, mutation detection, DNA assembly, DNA sequencing, and other aspects. Thermophilic and thermostable enzymes from hyperthermophiles that thrive in the high-temperature (above 80°C) environments have provided an important pool of useful enzymes as biotechnological reagents. Similar to other organisms, each hyperthermophile harbors at least one DNA ligase. In this review, we summarize recent progress on structural and biochemical properties of thermostable DNA ligases from hyperthermophiles, focusing on similarities and differences between DNA ligases from hyperthermophilic bacteria and archaea, and between these thermostable DNA ligases and non-thermostable homologs. Additionally, altered thermostable DNA ligases are discussed. Possessing improved fidelity or thermostability compared to the wild-type enzymes, they could be potential DNA ligases for biotechnology in the future. Importantly, we also describe current applications of thermostable DNA ligases from hyperthermophiles in biotechnology.

## Introduction

**Figure 1:**
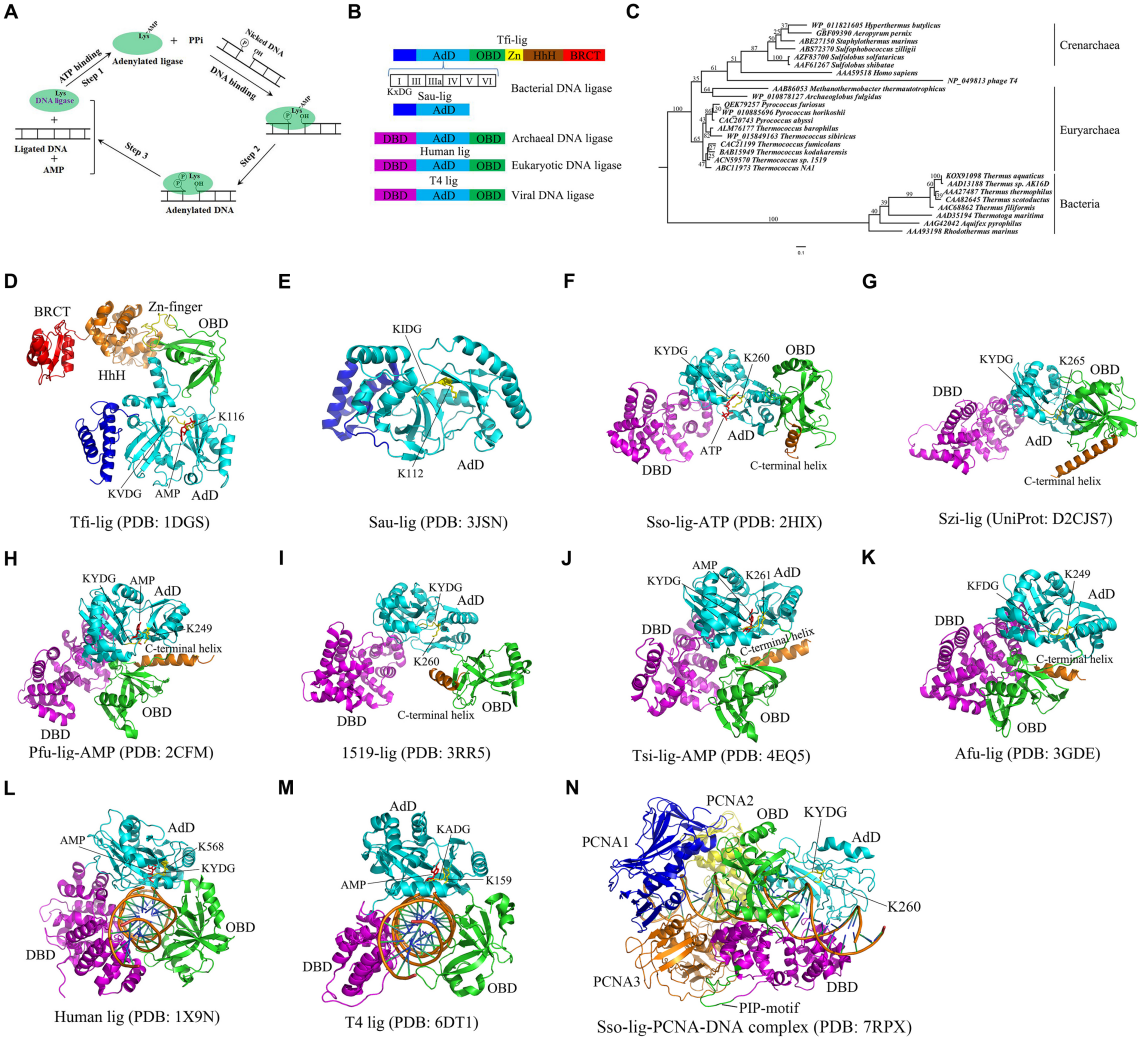
Catalytic mechanism, domain structures, phylogenetic analyses and crystal structures of DNA ligase. **(A)** Catalytic mechanism of DNA ligase. Note that ATP is used as a cofactor to clarify three-step mechanism of DNA ligase. NAD^+^ -dependent DNA ligase utilizes similar catalytic mechanism. **(B)** Domain structures of DNA ligases from bacteria, eukaryotes, archaea and viruses. Six motifs (I, III, IIIa, IV, V and VI) are present in the AdD of DNA ligases. The conserved “KxDG” is present in motif I. **(C)** Phylogenetic analyses of the reported thermostable DNA ligases from archaea, bacteria, eukaryotes and virus. **(D)** Crystal structure of Tfi*-*lig (*T. filiformis* DNA ligase). **(E)** Crystal structure of the truncated Sau-lig (*S. aureus* DNA ligase). **(F)** Crystal structure of Sso-lig (*S. solfataricus* DNA ligase) with ATP. **(G)** Crystal structure of Szi-lig (*S. zilligii* DNA ligase). **(H)** Crystal structure of Pfu-lig (*P. furiosus* DNA ligase) with AMP. **(I)** Crystal structure of 1519-lig (*Thermococcus* sp. 1519 DNA ligase). **(J)** Crystal structure of Tsi*-*lig (*T. sibiricus* DNA ligase) with AMP. **(K)** Crystal structure of Afu-lig (*A. fulgidus* DNA ligase). **(L)** Crystal structure of human DNA ligase I bound with DNA. **(M)** Crystal structure of T4-lig (T4 DNA ligase) bound with DNA. The structures of *S. aureus*, human and T4 DNA ligases were used for a comparison. **(N)** The Sso-lig-DNA-PCNA complex.

DNA ligase is one of essential enzymes involved in DNA replication, repair and recombination *in vivo*, thus playing important roles in maintaining genomic integrity. DNA ligase is widespread in bacteria, archaea, eukaryotes, and even viruses. All the reported DNA ligases employ a common three-step mechanism to form a phosphodiester bond using either ATP or NAD^+^ as a high-energy cofactor (Figure 1A; Williamson and Leiros, 2019; Williamson and Leiros, 2020). The first step is a nucleophilic attack on 5′-phosphate of ATP or NAD^+^ cofactor by the highly conserved lysine in DNA ligase, thus yielding the ligase-AMP intermediate and releasing PPi. The second step is to transfer AMP from the adenylated ligase to the 5′-phosphate of one strand, thereby leading to the formation of the adenylated DNA intermediate. Next, the third step is to join the DNA strands by forming a phosphodiester bond between the 5′-phosphate group of the adenylated DNA strand and the 3′-hydroxyl group of the second strand, thus releasing AMP (Williamson and Leiros, 2019). Based on their cofactor utilization specificity, DNA ligases are divided into two families: ATP-dependent DNA ligases and NAD^+^ -dependent DNA ligases. While ATP-dependent DNA ligases are typically present in archaea, eukaryotes, and viruses, including bacteriophages, NAD^+^ -dependent DNA ligases are predominately found in bacteria and some eukaryotic viruses.

All the reported DNA ligases possess the adenylation domain (AdD) and oligomer-binding domain (OBD) (Figure 1B), which are responsible for the formation of the ligase-AMP intermediate and DNA binding, respectively. Interestingly, bacterial DNA ligases appear to harbor more conserved motifs than archaeal, eukaryotic and viral DNA ligases. For example, the DNA ligase from the hyperthermophilic bacterium *Thermus filiform* possesses a domain containing a Zn finger motif and a HhH (Helix-hairpin-Helix) motif, and a BRCT (BRCA1 C-terminus) domain in addition to AdD and OBD (Figure 1B; Lee et al., 2000). Notably, six conserved motifs (I, III, IIIa, IV, V, and VI) are present in the AdD in DNA ligase. The “KxDG” sequence is highly conserved in the conserved motif I of DNA ligase, among which the invariable residue lysine is essential for the formation of the ligase-AMP intermediate.

*In vitro*, DNA ligases are used for molecular cloning, DNA assembly, DNA sequencing and mutation detection associated with genetic diseases (Barany, 1991; Duckworth et al., 2023). The ATP-dependent bacteriophage T4 DNA ligase is one of the most commercial DNA ligases in molecular cloning since it has a strong ability to join cohesive fragments (Shi et al., 2018). Additionally, the predominant shortcoming of this ligase is that it is irreversibly inactivated when exposed to temperatures above 65°C, which confines its application in biotechnology requiring DNA ligation to low temperature. Fortunately, thermostable DNA ligases from hyperthermophiles that can ligate DNA at the elevated temperature and possess a strong thermostabililty have been characterized, which are well suited for biotechnological applications in high-temperature environments.

Hyperthermophiles thrive in high-temperature environments at a temperature range of 80°C to 100°C (Verma et al., 2022). At least one DNA ligase is encoded in the genome of each hyperthermophile. Sequence alignments show that thermostable DNA ligases from archaea and bacteria display low similarities, but they possess six conserved motifs present in all the reported DNA ligases. The phylogenetic relationship of the thermostable DNA ligases from bacteria and archaea is shown in the phylogenetic tree constructed from the DNA ligases from bacteria and archaea, human and the bacteriophage T4 (Figure 1C), demonstrating that the human and T4 DNA ligases display a closer phylogenetic relationship to thermostable archaeal DNA ligases than bacterial DNA ligases. Compared to non-thermostable DNA ligases, thermostable DNA ligases possess a higher fidelity, which can avoid errors for ligation-based molecular diagnostic techniques. Besides, thermostable DNA ligases exhibit a strong thermostability, retaining activity after multiple thermal cycles. Additionally, some thermostable DNA ligases, such as HiFi Taq DNA ligase, appear to exhibit more effective discrimination between correct and mismatched base pairs at either side of nicks. Currently, *Pyrococcus furiosus* DNA ligase, *Thermococcus* 9^o^N DNA ligase, and Ampligase are commercially available and active at the elevated temperature.

In this review, we summarize current progress on structural and biochemical characteristics of thermostable DNA ligases from hyperthermophilic bacteria and archaea, focusing on similarities and differences between thermostable DNA ligases and non-thermostable homologs, and between thermostable bacterial and archaeal DNA ligases. Additionally, altered thermostable DNA ligases with improved properties and their potential applications in biotechnology are discussed.

## Thermostable DNA ligases from hyperthermophilic bacteria

Currently, eight DNA ligases from hyperthermophilic bacteria have been biochemically or structurally characterized from *Thermus thermophilus* HB8 (Takahashi et al., 1984), *Thermus* sp. AK16D (Tong et al., 1999), *Thermus aquaticus* (Housby et al., 2000), *Rhodothermus marinus* (Housby et al., 2000), *Thermus scotoductus* (Housby et al., 2000), *Thermus filiformi* (Lee et al., 2000), *Aquifex pyrophilus* (Lim et al., 2001), and *Thermotoga maritima* (Le et al., 2010). The common characteristic of these thermostable bacterial DNA ligases is that they utilize NAD^+^ rather than ATP as a cofactor (Table 1).

**Table 1:** Comparison of biochemical characteristics of DNA ligases from hyperthermophilic bacteria and archaea.

Hyperthermophile		Optimal T^a^ (°C)	Optimal pH	Divalent ion requirement	Cofactor	PDB ID	Reference
Bacteria	*Thermus thermophilus* HB8	65 ~ 72	N.D.^b^	Mg^2+^ and Mn^2+^	NAD^+^	N.D.	Takahashi et al. (1984)
	*Thermus* sp. AK16D	N.D.	N.D.	Mg^2+^, Mn^2+^ and Ca^2+^	NAD^+^	N.D.	Tong et al. (1999)
	*Thermus aquaticus*	N.D.	N.D.	N.D.	N.D.	N.D.	Housby et al. (2000)
	*Rhodothermus marinus*	55	N.D.	N.D.	NAD^+^	N.D.	Housby et al. (2000)
	*Thermus scotoductus*	65	N.D.	N.D.	NAD^+^	N.D.	Housby et al. (2000)
	*Thermus filiformis*	N.D.	N.D.	N.D.	N.D.	1DGS	Lee et al. (2000)
	*Aquifex pyrophilus*	65	8.0 ~ 8.6	Mg^2+^ and Mn^2+^	NAD^+^	N.D.	Lim et al. (2001)
	*Thermotoga maritima*	60	8.0	Mg^2+^, Mn^2+^ and Ca^2+^	NAD^+^	N.D.	Le et al. (2010)
Archaea	*Methanobacterium thermoautotrophicum*	70	N.D.	N.D.	N.D.	N.D.	Sriskanda et al. (2000)
	*Thermococcus kodakaraensis*	100	8.0	Mg^2+^, Mn^2+^, Sr^2+^ and Ca^2+^	ATP and NAD^+^	N.D.	Nakatani et al. (2000)
	*Sulfolobus shibatae*	60 ~ 80	N.D.	Mn^2+^, Mg^2+^ and Ca^2+^	ATP and dATP	N.D.	Lai et al. (2002)
	*Aeropyrum pernix*	70	7.5	Mg^2+^, Mn^2+^, Ca^2+^ and Co^2+^	ADP and ATP	N.D.	Jeon and Ishikawa (2003)
	*Thermococcus fumicolans*	65	7.0	Mg^2+^	ATP and NAD^+^	N.D.	Rolland et al. (2004)
	*Pyrococcus horikoshii*	70 ~ 90	N.D.	N.D.	ATP	N.D.	Keppetipola and Shuman, 2005
	*Pyrococcus furiosus*	N.D.	N.D.	N.D.	N.D.	3GDE	Nishida et al. (2006)
	*Thermococcus* sp. NA1	80	7.5	Mg^2+^ and Zn^2+^	ATP and NAD^+^	N.D.	Kim et al. (2006)
	*Sulfolobus solfataricus*	N.D.	N.D.	N.D.	N.D.	2HIX, 7RPX	Pascal et al. (2006) and Sverzhinsky et al. (2022)
	*Staphylothermus marinus*	N.D.	N.D.	Mg^2+^ and Mn^2+^	N.D.	N.D.	Seo et al. (2007)
	*Sulfophobococcus zilligii*	N.D.	N.D.	N.D.	ADP, GTP and ATP	D2CJS7^c^	Sun et al. (2008)
	*Archaeoglobus fulgidus*	N.D.	N.D.	N.D.	N.D.	2CFM	Kim et al. (2009)
	*Thermococcus sibiricus*	N.D.	N.D.	N.D.	N.D.	4EQ5	Petrova T. E. et al. (2012)
	*Thermococcus* sp. 1,519	70	7.0 ~ 10.5	Mg^2+^	ATP	3RR5	Petrova T. et al. (2012)
	*Hyperthermus butylicus*	75	8.0	Mg^2+^ and Mn^2+^	ADP, GTP and ATP	N.D.	Kim et al. (2013)
	*Pyrococcus abyssi*	70	N.D.	N.D.	N.D.	N.D.	Oscorbin et al. (2015)
	*Thermococcus barophilus* Ch5	65 ~ 70	6.0 ~ 9.0	Mn^2+^, Mg^2+^ and Ca^2+^	ATP and UTP	N.D.	Shi et al. (2019)

a T, Temperature.

b N.D., Not determined.

c The crystal structure of the *S. zilligii* DNA ligase was solved, but no PDB accession number is given.

Besides, these four DNA ligases from *T. thermophilus* HB8, *Thermus* sp. AK16D, *A. pyrophilus*, and *T. maritima* display maximum activity in the presence of Mg^2+^ or Mn^2+^. In addition to Mg^2+^ or Mn^2+^, Ca^2+^ can also stimulate the ligation activities of the *Thermus* sp. AK16D and *T. maritima* DNA ligases. The optimal temperatures of these thermostable bacterial DNA ligases are at a temperature range of 55°C ~ 72°C, demonstrating that they are thermophilic. Additionally, the DNA ligases from *A. pyrophilus* and *T. maritima* retain their ligation activity after heating at 95°C for 30 min and 60 min, respectively, thus showing that they are thermostable DNA ligases. However, the *R. marinus* and *T. scotoductus* DNA ligases lose their activity after heating at 91°C for 7 min and 26 min, respectively. Besides, the *T. maritima* and *Thermus* sp. AK16D DNA ligases exhibit the highest ligation activity at pH 8.0.

The *T. thermophilus* DNA ligase is one of the well-characterized DNA ligases from hyperthermophilic bacteria (Takahashi et al., 1984; Luo et al., 1996; Luo and Barany, 1996; Muerhoff et al., 2004; Wang et al., 2020). Compared to the mesophilic ATP-dependent DNA ligases, the *T. thermophilus* DNA ligase possesses three distinct characteristics. Firstly, the optimal temperature of the *T. thermophilus* DNA ligase was estimated to be approximately 65°C instead of 37°C. Secondly, the enzyme possesses strong thermostability. Thirdly, the enzyme displays a higher fidelity than the T4 DNA ligase.

The crystal structure of the NAD^+^ -dependent *T. filiformi* DNA ligase was solved (Figure 1D), displaying a highly modular architecture that comprises four domains including the AdD (domain 1), the OBD (domain 2), the domain 3 harboring a zinc finger and a HhH motif, and BRCT (domain 4). In contrast to the truncated *S. aureus* DNA ligase lacking OBD (Figure 1E; Han et al., 2009), the *T. filiformi* DNA ligase harbors the OBD, which is involved in DNA binding DNA. Besides to the OBD (domain 2), the zinc finger motif (subdomain 3a) and the HhH motif domain (subdomain 3b), which are all known to bind to nucleic acids (Trasviña-Arenas et al., 2021; Hajdu et al., 2023), are present in the *T. filiformi* DNA ligase, demonstrating a unique organization and spatial arrangement of these domains. Additionally, the *T. filiformi* DNA ligase has a BRCT domain with much mobility in the open conformation, which might act as a gate to regulate DNA binding and release. Overall, the structure of the *T. filiformi* DNA ligase provides insight into the understanding of domain organization, catalytic mechanism and evolution of covalent nucleotidyl transferases, which might be generally applicable to eukaryotic DNA ligases.

Mutational analyzes show that only the AdD of the *T. filiformis* DNA ligase is responsible for forming the ligase-AMP complex (Jeon et al., 2004). The OBD, the zinc finger and HhH motif in the domain 3 of the *T. filiformis* DNA ligase are essential for forming the ligase-DNA complex. However, the BRCT domain of the *T. filiformis* DNA ligase is not essential for the enzyme activity. In contrast, substantial loss of ligation activity was observed for the mutant with the deletion of the BRCT domain of the *Thermus* sp. AK16D DNA ligase (Feng et al., 2004). However, the mutant can still form a ligase-NMP intermediate rather than NMP-DNA accumulation, suggesting that the BRCT domain is involved in the substrate adenylation step. Further mutational analysis shows that the mutation of G617 in the *Thermus* sp. AK16D DNA ligase to Ile resulted in a low ligation activity and caused this enzyme to be weakly adenylated (Feng et al., 2004), suggesting that residue G617 in the BRCT domain plays a role in the enzyme adenylation step.

## Thermostable DNA ligases from hyperthermophilic archaea

Seventeen DNA ligases have been reported from hyperthermophilic archaea to date, including *Methanobacterium thermoautotrophicum* (Sriskanda et al., 2000), *Thermococcus kodakaraensis* (Nakatani et al., 2000), *Sulfolobus shibatae* (Lai et al., 2002), *Aeropyrum pernix* (Jeon and Ishikawa, 2003), *Thermococcus fumicolans* (Rolland et al., 2004), *Pyrococcus horikoshii* (Keppetipola and Shuman, 2005), *P. furiosus* (Nishida et al., 2006), *Thermococcus* sp. NA1 (Kim et al., 2006), *Sulfolobus solfataricus* (Pascal et al., 2006), *Staphylothermus marinus* (Seo et al., 2007), *Sulfophobococcus zilligii* (Sun et al., 2008), *Archaeoglobus fulgidus* (Kim et al., 2009), *Thermococcus sibiricus* (Petrova T. E. et al., 2012), *Thermococcus* sp. 1519 (Petrova T. et al., 2012), *Hyperthermus butylicus* (Kim et al., 2013), *Pyrococcus abyssi* (Oscorbin et al., 2015), and *Thermococcus barophilus* Ch5 (Shi et al., 2019). Comparison of biochemical characteristics of thermostable archaeal and bacterial DNA ligases is summarized in Table 1, highlighting their similar and different properties.

In contrast to DNA ligases from hyperthermophilic bacteria that specifically use NAD^+^ as a cofactor, DNA ligases from hyperthermophilic archaea preferably utilize ATP as a cofactor. In addition to ATP, the DNA ligases from *T. kodakaraensis*, *T. fumicolans*, and *Thermococcus* sp. NA1 can also use NAD^+^ for their ligation activity. Interestingly, ADP can be used as a cofactor for DNA ligases from *H. butylicus*, *S. zilligii*, and *A. pernix* to join DNA. Besides ATP and NAD^+^, GTP is also a cofactor for the DNA ligases from *H. butylicus* and *S. zilligii*. Additionally, dATP and UTP can be used by the DNA ligase from *S. shibatae*. Interestingly, no extra ATP is needed for the recombinant *T. barophilus* Ch5 DNA ligase to ligate DNA since the enzyme is already adenylated after purification, which can reduce the cost of its application in biotechnology.

As observed for DNA ligases from hyperthermophilic bacteria, DNA ligases from hyperthermophilic archaea display maximum activity at the elevated temperature (65°C ~ 100°C). Besides, the DNA ligases from hyperthermophilic *S. marinus*, *A. pernix*, and *T. barophilus* Ch5 retain ligation activity after heating 100°C for at least 1 h, suggesting that their thermostablilty is higher than that of DNA ligases from hyperthermophilic bacteria. In contrast, the DNA ligases from *P. abyssi* and *Thermococcus* sp. 1519 display a lower thermostability than that of the DNA ligases from *T. maritima* and *A. pyrophilus.* Moreover, the *T. kodakaraensis* DNA ligase displays a nick-sealing activity at 100°C, demonstrating that it possesses the highest thermophilicity.

The crystal structures of six thermostable DNA ligases from hyperthermophilic archaea have been solved to date, comprising three domains: AdD, OBD, and the N-terminal DNA-binding domain (DBD) (Figure 1B), which includes *S. solfataricus* (Figure 1F; Pascal et al., 2006), *S. zilligii* (Figure 1G; Supangat et al., 2010), *P. furiosus* (Figure 1H; Nishida et al., 2006), *Thermococcus* sp. 1519 (Figure 1I; Petrova T. et al., 2012), *T. sibiricus* (Figure 1J; Petrova T. E. et al., 2012), and *A. fulgidus* (Figure 1K; Kim et al., 2009). In contrast to the *T. filiformi* DNA ligase structure, thermostable DNA ligases from these hyperthermophilic archaea lack the Zn-finger motif, HhH motif, and BRCT domain, but possess the DBD. Interestingly, the N-terminal DBD is absent in bacterial DNA ligase, but only present in the eukaryotic and archaeal DNA ligases, which might participate in distorting the DNA substrate and maintaining an active conformation of the catalytic core. Similar to the *T. filiformi* DNA ligase structure, these archaeal DNA ligases harbor the AdD and OBD, but their conformation varies. Although the structures of thermostable DNA ligases resemble the DNA-bound structures of the human DNA ligases I (Figure 1L) and T4 DNA ligase (Figure 1M; Pascal et al., 2004; Shi et al., 2018), which are distinct from the structures of bacterial DNA ligases, their domain arrangements differ substantially. The conformational flexibility might be critical for ligating DNA of thermostable DNA ligases.

The open and extended conformations of AdD and OBD were captured in the *S. solfataricus* DNA ligase, where its OBD was turned away from the AdD (Figure 1F). In contrast, an intermediate conformation is found in the *Thermococcus* sp.1519 ligase structure (Figure 1I), where its OBD was rotated anticlockwise around the AdD by ∼ 90^o^, which sharply contrasts with the open extended conformation in the *S. solfataricus* DNA ligase. Besides, the structures of the DNA ligases from *P. furiosus* (Figure 1H), *T. sibiricus* and *A. fulgidus* adopted a closed conformation that was yielded with a further 120^o^ rotation of the OBD (Figures 1J,K). Interestingly, their closed conformation might be stabilized with a C-terminal helix in these structures via regulating several ionic interactions between the AdD and the OBD in *P. furiosus* DNA ligase (Figure 1H).

*S. solfataricus* possesses three proliferating cell nuclear antigen (PCNA 1, 2 and 3) proteins, which sharply contrasts with *P. furiosus* harboring the homotrimeric PCNA. Each subunit of the *S. solfataricus* PCNA interacts with a specific enzyme, among which the PCNA3 client enzyme is the ligase (Dionne et al., 2003). Biochemical data show that the *S. solfataricus* DNA ligase activity was stimulated by the heterotrimeric *S. solfataricus* PCNA by enhancing ligase encirclement at nicked DNA (Dionne et al., 2003; Pascal et al., 2006), which was confirmed in a negative stain electron microscopy structure of the *P. furiosus* DNA ligase in complex with a nicked, non-ligatable DNA and homotrimeric PCNA by single-particle analysis (Mayanagi et al., 2009). Recently, Sverzhinsky et al. (2022) solved the cryo-EM structure of the complex of the *S. solfataricus* DNA ligase, DNA, and the heterotrimeric PCNA (Figure 1N), demonstrating that the canonical PCNA-interacting peptide (PIP) motif in the DBD of the ligases interacts with the inter-domain connecting loop of PCNA3, which provide support for this stimulation of the ligase activity by the heterotrimeric PCNA.

## Thermostable DNA ligases: ligase chain rection for detection of single nucleotide polymorphism

**Figure 2:**
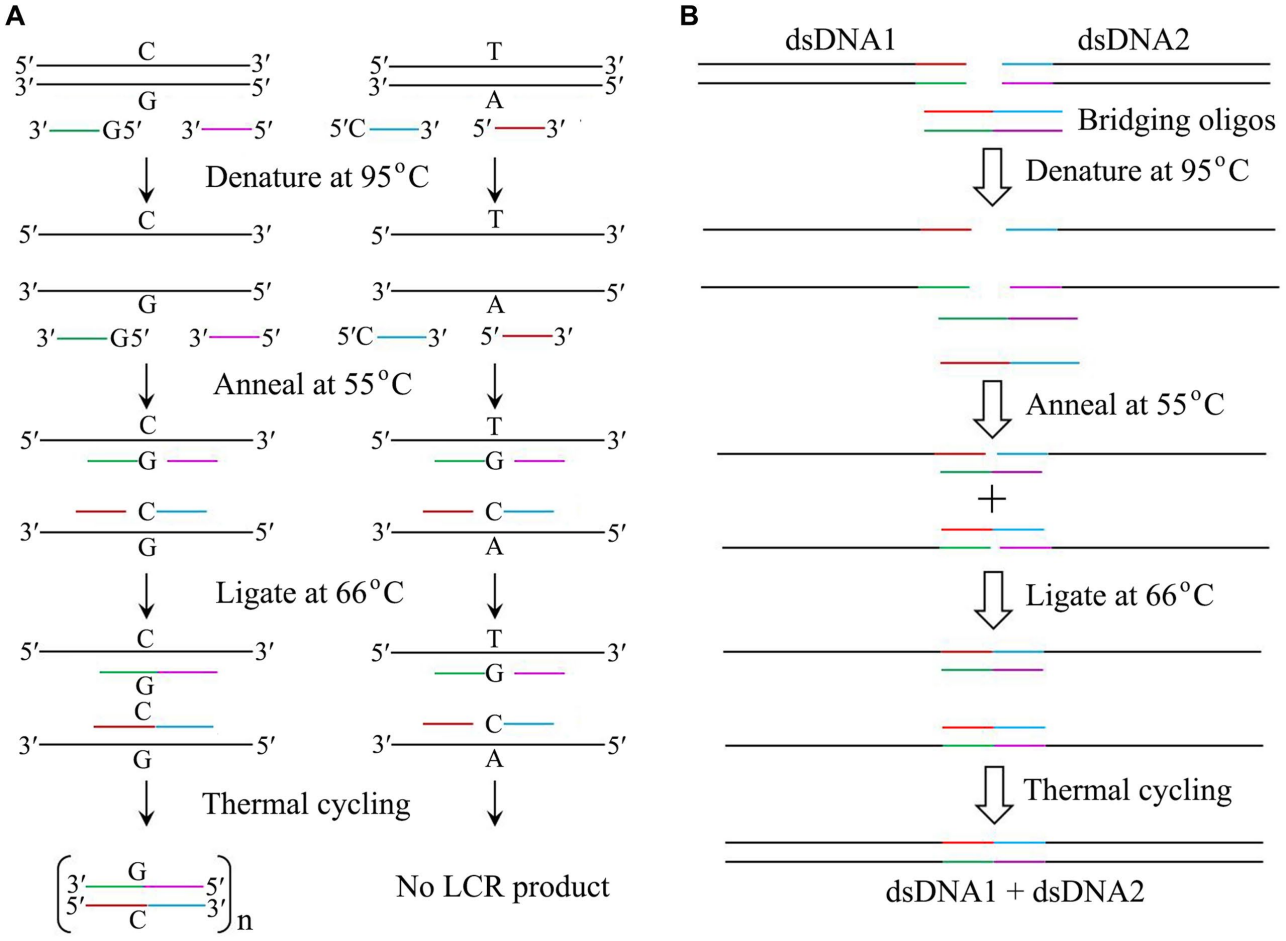
Application of thermostable DNA ligases from hyperthermophiles. **(A)** Mutation detection by LCR catalyzed by thermostable DNA ligases. Note that both probes of 5′-G-oligo and 5′-C-oligo are phosphorylated. **(B)** DNA assembly by LCR catalyzed by thermostable DNA ligases. The details are in text.

Like PCR, ligase chain rection (LCR) mediated by thermostable DNA ligase can be used to amplify DNA to detect a single base substitution since this ligase can specifically seal two adjacent oligonucleotides complementary to a DNA template strand containing a perfect base pair rather than a single base mismatch (Gibriel and Adel, 2017; Liang et al., 2022). LCR is usually performed by a thermal cycling of denaturing the target dsDNA at 95°C, annealing the probes with the corresponding denatured template at 55°C, and ligating the nicks created between the two probes at 66°C by thermostable DNA ligase (Figure 2A). If the single base mismatch is present in the target dsDNA, no ligation product would be observed. Thus, LCR is a powerful method for the detection of single base mutation in single nucleotide polymorphism (SNP).

Thermostable DNA ligase is a cornerstone for LCR due to its high fidelity and strong thermostability, thus allowing the useful DNA-based diagnostic tests for inherited diseases to be performed in clinical laboratories (Liu et al., 2020; Malik et al., 2021; Zhang et al., 2021; Li et al., 2022). The thermostable DNA ligases from *T. thermophilus* and *T. aquaticus* display the higher ligation fidelity than T4 DNA ligase (Barany, 1991; Luo et al., 1996), and have been commercially used for LCR to date. Currently, LCR mediated by thermostable DNA ligase has been employed to detect point mutations associated with genetic diseases.

Besides LCR, ligase detection reaction (LDR), ligation amplification reaction (LAR), and gap-LCR have also been developed with thermostable DNA ligases (Wu and Wallace, 1989; Abravaya et al., 1995; Li et al., 2022). Additionally, thermostable DNA ligases can also be used for rolling-circle amplification (RCA) in the presence of padlock probes, which is an alternative method for detecting SNP (Yan et al., 2023). Modification of LCR, gap-LCR was developed by filling in a gap between annealed probes by DNA polymerase to reduce the background generated by target-independent, blunt-end ligation. To date, it has been confirmed that gap-LCR is useful for detection of a mutation in the reverse transcriptase gene of HIV that confers AZT resistance (Wu and Wallace, 1989; Abravaya et al., 1995).

## Thermostable DNA ligases: LCR for DNA assembly

Since establishing a highly efficient DNA assembly method is significantly essential for automated high-throughput DNA assembly, De Kok et al. (2014) reported a one-step, scarless DNA assembly via LCR involving a thermostable ligase by using single-stranded bridging oligos complementary to the ends of target DNA. DNA assembly is usually completed by a thermal cycling of denaturing two substrates and two bridging oligos at 95°C, annealing the bridging oligo with the target dsDNA at 55°C, and ligating the nicks created between the bridging oligo and the target dsDNA at 66°C by thermostable DNA ligase (Figure 2B). Compared to Gibson isothermal assembly (Gibson et al., 2009), DNA assembly mediated by LCR is a rapid and reliable method with a cheap, rapid, and convenient workflow. Therefore, LCR has become a powerful method for both manual and automated high-throughput DNA assembly.

Recently, an effective DNA assembly method was reported using thermostable exonuclease and ligase “(DATEL)” including *T. aquaticus* DNA ligase, thermal exonucleases (*T. aquaticus* and *P. furiosus* DNA polymerases; Kang et al., 2018). Due to rapid assembly (2 ~ 10 DNA fragments per 1 ~ 2 h), high accuracy (between 74 and 100%) and the simple operation system, DATEL displays a great potential for high-throughput assembly of DNA fragments, which will greatly promote the rapid development of metabolic engineering and synthetic biology.

## Thermostable DNA ligases: directed evolution

Directed evolution is an important method to engineer enzyme mutants. The thermostable DNA ligase from *T. maritima* can be used to mediate PCR production of circular plasmid (PPCP) that is catalyzed by the *T. aquaticus* DNA polymerase (Le et al., 2013). This PPCP method has an important application in directed evolution by allowing one-step construction of mutagenesis libraries through *in situ* error-prone PCR. For example, random mutagenesis libraries of a xylanase gene and two cellulase genes have been created by this PPCP method mediated by the *T. maritima* DNA ligase. Therefore, *in situ* error-prone PPCP mediated by thermostable DNA ligase is useful for generating random mutagenesis libraries for directed evolution.

## Altered thermostable DNA ligases with the improved properties

Protein engineering is a powerful method for constructing enzyme mutants with the enhanced catalytic properties. The *P. furiosus* DNA ligase is the first reported thermostable DNA ligase that has been altered (Nishida et al., 2006). Besides, DNA ligases from *Thermus* sp. AK16D, *Thermococcus* sp. 1,519 and *T. thermophilus* were also targeted for engineering mutants with better properties (Luo et al., 1996; Feng, 2007; Modarres et al., 2015), such as increased fidelity and thermostability.

Compared to the wild-type protein, the D540R mutant of the *P. furiosus* DNA ligase displayed the nick-joining activity at a broaden temperature range (20°C ~ 80°C) (Tanabe et al., 2014). Besides, the biophysics experiments confirmed that the D540R mutant exhibits the increased binding of the nicked DNA substrate and formation of the covalent ligase-AMP intermediate (Tanabe et al., 2012). Additionally, a further increased nick-sealing activity was observed for the engineered *P. furiosus* DNA ligase mutants harboring the D540R mutation plus K554A/K558A mutations or the D540R mutation and a deletion of the final four residues of the C-terminal helix.

Using molecular modeling and simulations, Modarres et al. (2015) predicted the substitutions that can increase thermostability of the DNA ligase from *Thermococcus* sp. 1,519 and identified its thermosensitive regions. By optimizing the charged groups on the surface in the thermosensitive regions by introducing the selected mutations (A287K, G304D, S364I, and A387K), the remarkable and additive increase of thermostability of the enzyme was observed relative to the wild-type protein. Thus, the altered *Thermococcus* sp. 1519 DNA ligase with increased thermostability might provide potential DNA ligases used for biotechnology.

LCR and LDR are the powerful methods for the detection of SNPs, which require a thermostable DNA ligase with high fidelity. Notably, the mutations at residue K294 in the *T. thermophilus* DNA ligase to be Arg and Pro increased 4-fold fidelity and 11-fold fidelity in addition to still retaining the nick-sealing activities (Luo et al., 1996), respectively. Additionally, the mutations of D286E/G287A/V289I/K291R resulted in the enhanced ligation fidelity of the *Thermus* sp. AK16D DNA ligase (Feng, 2007). Thus, these altered DNA ligase mutants with high-fidelity are potential targets for LCR and LDR.

## Conclusion and future directions

Thermostable DNA ligases from hyperthermophiles display structural and biochemical properties distinct from those of non-thermostable orthologs. Several DNA ligase mutants from *P. furiosus*, *Thermococcus* sp. 1519, and *T. thermophilus* with improved properties have been engineered to date. However, the thermostable DNA ligase mutants with superior properties need to be engineered by directed evolution to meet the demands of biotechnological development.

Currently, no crystal structure of the complex of thermostable DNA ligase with DNA has been solved. It will be worthwhile to solve the structures of the thermostable DNA ligase complexes with DNA, which might provide insight into understanding structural and functional similarities and differences between thermostable DNA ligases and non-thermostable orthologs. Additionally, the relationship between protein dynamics and catalysis of thermostable DNA ligases at the elevated temperature needs to be investigated.

## Author contributions

JS made Table 1 and wrote this manuscript; PO wrote and revised this manuscript; PC made Figure 2; LZ designed, wrote, and revised this manuscript. All authors approved it for publication.

## Funding

This is funded by Natural Science Foundation of Jiangsu Province (No. BK20191219), and High Level Talent Support Program of Yangzhou University the Academic Leader of Middle and Young People of Yangzhou University Grant to LZ.

## Conflict of interest

The authors declare that the research was conducted in the absence of any commercial or financial relationships that could be construed as a potential conflict of interest.

## Publisher’s note

All claims expressed in this article are solely those of the authors and do not necessarily represent those of their affiliated organizations, or those of the publisher, the editors and the reviewers. Any product that may be evaluated in this article, or claim that may be made by its manufacturer, is not guaranteed or endorsed by the publisher.
